# Vessel density and En-face segmentation of optical coherence tomography angiography to analyse corneal vascularisation in an animal model

**DOI:** 10.1186/s40662-018-0128-8

**Published:** 2019-01-08

**Authors:** Kavya Devarajan, Wen Di Lee, Hon Shing Ong, Nyein C. Lwin, Jacqueline Chua, Leopold Schmetterer, Jodhbir S. Mehta, Marcus Ang

**Affiliations:** 1 Singapore Eye Research Institute, Singapore, Singapore; 2 Singapore National Eye Center, Singapore, Singapore; 3 Eye-ACP, Duke-NUS Graduate Medical School, Singapore, Singapore; 4 Department of Clinical Pharmacology, Medical University of Vienna, Vienna, Austria; 5 Center for Medical Physics and Biomedical Engineering, Medical University of Vienna, Vienna, Austria; 6 Nanyang Technological University, Singapore, Singapore

**Keywords:** OCTA, Corneal vascularisation, Optical micro angiography, Split-spectrum amplitude decorrelation angiography, Vessel density, Anterior segment, En-face OCTA

## Abstract

**Background:**

Optical coherence tomography angiography (OCTA) is a novel non-invasive angiography technology that has recently been extensively studied for its utility in anterior segment imaging. In this study, we compared a split-spectrum amplitude decorrelation angiography (SSADA) OCTA and an optical micro-angiography (OMAG SD) OCTA system to current angiographic technique, indocyanine green angiography (ICGA), to assess corneal vascularisation in an animal model.

**Methods:**

We imaged 16 rabbits, (one eye per animal) with corneal vascularisation using SSADA OCTA (AngioVue; Optovue Inc., USA), OMAG OCTA (Angioscan; RS-3000 Nidek Co. Ltd., Japan) and ICGA in the same region of interest of the cornea at successive time-points. We then analysed all scanned images for vessel density measurements and used paired t-tests and Bland-Altman plots to examine for significant differences. The en-face segmentation images from each of the OCTA scans were also extracted and were matched at every 50 μm segmentation to be compared for vessel density at the respective depths.

**Results:**

Bland-Altman plots revealed a good agreement between all three imaging techniques (*P* > 0.05) for all vessel density measurements computed, and the ranges of 95% limit of agreement were acceptable from a clinical perspective. No significant difference was reported, with ICGA (μ = 16.52 ± 8.94%) being more comparable to the OMAG OCTA (μ = 16.23 ± 9.51%; *p* = 0.50) than the SSADA OCTA (μ = 17.09 ± 7.34%; *p* = 0.33) system. Also, a good correlation value (*r* > 0.9) was obtained when comparing the vessel density measurements of the en-face segmentations between the OCTA systems.

**Conclusions:**

Comparable vessel density quantification between the two OCTA systems, and with ICGA was obtained. Segmentation analysis of the vasculature at different depths showed varied performance in the two OCTA systems relative to each other. The implications of the study may help to aid in the development of better OCTA algorithms for the anterior segment and its use in clinical translational research.

## Background

Corneal vascularisation is a sight-threatening condition that involves the pathological ingrowth of blood vessels into the typically avascular cornea, in response to inflammation, infection, trauma or hypoxia [1, 2]. It impairs light transmission, promotes scar formation and results in persistent inflammation thereby affecting visual acuity [3]. Conventional treatment options and prevention of visual loss in patients with corneal vascularisation remains a key challenge for clinicians [2]. There have been recent attempts to achieve novel drug therapies targeting the molecular mechanisms of corneal vascularisation. However, the ability to quantitatively assess or objectively evaluate corneal vascularisation before and after any intervention is still limited [4]. Therefore, a reliable imaging system to evaluate and quantify corneal vascularisation and its response to treatment is much needed [5].

Corneal vascularisation is usually assessed by analysing images of the cornea taken by slit lamp biomicroscopy. However, they do not represent an objective representation of the corneal vasculature, especially in the presence of corneal scars, deposits or oedema [2, 4]. Indocyanine green angiography (ICGA) and fluorescein angiography (FA) have been shown to delineate corneal vessels and detect areas of corneal vascularisation through intravenous dye injections [6]. Among these, ICG (Indocyanine-green) is larger, more protein-bound than fluorescein and retains in the vessels for a longer duration, attributing for better vessel delineation [7]. ICGA is also shown to provide better image quality than FA [4]. Yet, both the angiography methods measure the vascularized area only in two dimensions and can be associated with adverse systemic side-effects [5].

Optical coherence tomography angiography (OCTA) is an emerging diagnostic tool for the anterior segment vasculature that overcomes the limitations of conventional techniques by providing three-dimensional structural and vascular information by non-invasive means [8, 9]. This technology has been recently adapted to image the anterior-segment of the eye and determined to be superior over conventional imaging modalities [8, 10–12].

However, the quantification and improvement of automated segmentation algorithms is still an active area of research and development in OCTA [13, 14]. OCTA for the retina is known to suffer from poor anatomical segmentation and pathology localisation due to the under-performance of automatic segmentation algorithms in diseased conditions thus making it difficult to interpret [13–15]. Moreover, OCTA is currently challenged by methodical and technical issues, such as vessel duplication, residual motion line artefacts and vessel discontinuity that are not present in conventional angiography [12]. Without suitable eye-tracker systems for the cornea, orthogonal line artefacts more predominantly occur during patient movement in the anterior-segment where the system fails and gives a false signal at all positions in the slow axis. The above disadvantages of OCTA is expected to lead amplified segmentation errors during cornea vasculature scanning as the application of the system at the anterior segment is yet to be realised [12, 16].

Although there are various OCTA systems available in the market that have been manipulated to image the anterior segment, there are limited studies comparing these systems that highlight the constraints and advantages for this purpose. The angiography algorithm in various OCTA systems may differ in the penetration depth and enhancement of fine vasculature resolution that can offer different diagnostic sensitivities [7]. This information can be useful when deciding the type of OCTA system to use in future research studies or clinical applications. Previously, we compared the systems for clinical investigation of corneal vascularisation and evaluated vessel density measurements in human eyes in a small pilot study [10]. However, it is still necessary to compare the systems to ICGA, to study the effects on the segmentation in animal models, as they provide good controls for corneal vascularisation. Furthermore, there are no studies thus far that have compared the segmentation methods of different OCTA systems [10]. Thus, there is a need to assess and compare the capabilities and limitations of the OCTA systems available for imaging corneal vascularisation.

In this study, we compared two OCTA systems that employ different algorithms in spectral-domain OCT for angiography acquisition, i.e., optical micro-angiography (OMAG OCTA Angioscan; RS 3000 Nidek Co. Ltd., Japan) and split-spectrum amplitude decorrelation (SSADA, AngioVue; Optovue Inc., USA) with ICGA, to image corneal vascularisation in a rabbit model.

## Methods

### OCTA systems

Generally, the working principle of OCTA systems can be divided into three categories: (i) angiography based on both the amplitude and phase of OCT signal, i.e., complex signal; (ii) angiography based on the amplitude of OCT signal, and (iii) angiography based on the phase of OCT signal [17] . The split spectrum amplitude decorrelation algorithm (SSADA) is based on the amplitude of the OCT signal that is enhanced for signal-to-noise ratio and flow detection by employing split-spectrum averaging algorithm. The algorithm is based on the splitting of the full OCT spectrum into several narrower bands from which the inter-B-scan decorrelation is computed using the spectral bands separately and then averaged [18]. It has a transverse resolution of 15 μm and axial resolution of 3 μm and acquires 70,000 A scans/sec using a light source centred on 840 nm with a beam width of 22 μm [10]. On the other hand, the OMAG OCTA system uses both amplitude and phase information as a complex signal to generate the angiography signal, allowing higher sensitivity to image vascular details. It uses a modified Hilbert transform to separate the moving scattering signals from the static background [18]. The system has a lateral resolution of 20 μm and axial resolution of 7 μm and captures 53,000 A scans/s using a light source centred at 880 nm [10].

### Image acquisition

The study was conducted on sixteen clinically healthy New Zealand white adult rabbits of either sex between the age group 12–15 weeks and weighing between 2.5–3.5 kg. Routine clinical evaluation and pre-operative ophthalmic examination of both eyes on all the animals was done prior to the experiment.

We performed consecutive follow-up imaging in rabbits with an established model of corneal vascularisation. The right eye of each rabbit underwent corneal suturing under general anaesthesia consisting intramuscular xylazine HCl (5 mg/kg) and ketamine HCl (50 mg/kg), supplemented with topical anaesthesia (0.4% oxybuprocaine HCl). The method of suture technique was described previously [5]; 10–0 non-absorbable nylon sutures (B. Braun Surgical SA, Spain) were placed at mid-stromal depth in the superior part of the cornea, in an inverted triangle fashion, step-wise to direct the growth of vessels centralized on the cornea. Antibiotic eye drops (tobramycin ophthalmic eye drops 0.3%, Alcon Labs Inc., Texas, USA) were applied twice daily throughout the follow-up period. The sutured eyes were followed up for two weeks after the suture-induced experiment when adequate development of induced corneal vascularisation was observed for the disease model. New vessels in the cornea started growing from the first week and reached the central cornea at the second week. The stitches were left intact to prevent any interruption or removal of the vessel growth inducing factor in the disease model.

The rabbits were imaged under anaesthesia with slit lamp photography (SLP), OMAG OCTA, SSADA OCTA and ICGA on a weekly basis throughout the follow-up period for two weeks. After which, histology studies were done, and the rabbits were then sacrificed.

Colour SLP images were captured using the digital slit-lamp camera (Righton MW50D, LED slit lamp, Miyagi, Japan) with a standard diffuse illumination (× 12 to × 36 magnification). For OCTA acquisition, the anterior segment lens was used with the AngioRetina scan protocol for the SSADA OCTA device and AngioMacula scan protocol for the OMAG OCTA device. In both the imaging acquisition software, the eye tracking and autofocus functions were deactivated. The lens was moved very close to the corneal surface before manual adjustments were made to the Z-motor positioning and focal length to achieve precise focus on the B-scan area of interest [10]. Anterior-segment scans using OCTA and ICGA centred on the corneal vasculature were evaluated for vessel density computations from week 1 and 2 follow-up time-points. A total of 32 images segmented at the whole B-scan depth (two time-point scans from 16 rabbits) were evaluated from each OCTA and ICGA system for vessel density comparison.

**Fig. 1:**
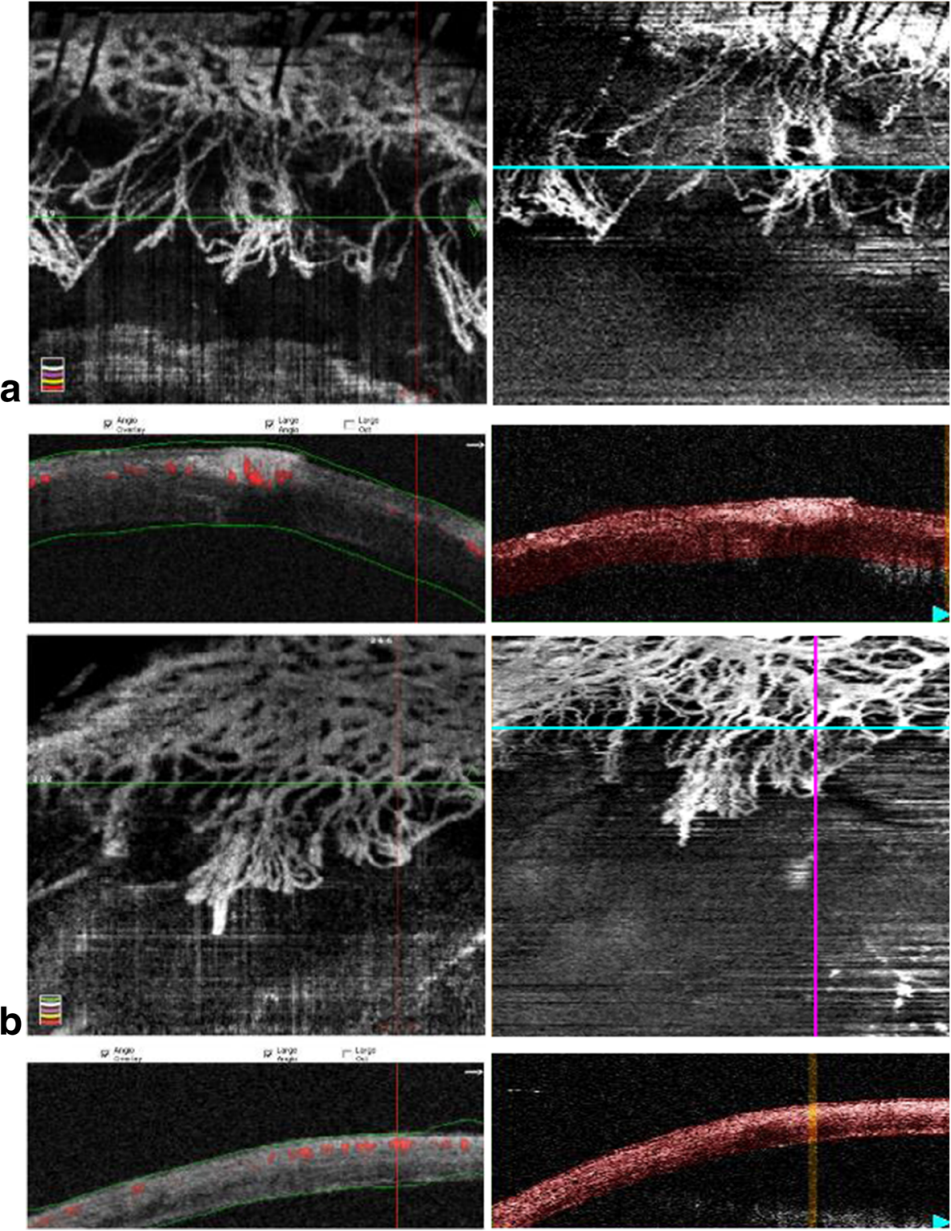
Representative examples of OCTA scans captured at (**a**) Week 2 and (**b**) Week 1 follow-up imaging. Example of OCTA images taken at Week 2 time-point (Fig. 1a) and Week 1 time-point (Fig. 1b) with whole cornea cross-sectional segmentation comparing SSADA OCTA (left) versus the OMAG OCTA system (right). The relatively higher image quality performance in the SSADA OCTA than the OMAG OCTA is observed. The highlighted red areas in the SSADA B-scans indicate the blood flow corresponding to the cross-sectional area marked by the horizontal green line in the en-face image. It is confirmed that the corneal vessels are present at the mid-stromal depth

**Fig. 2:**
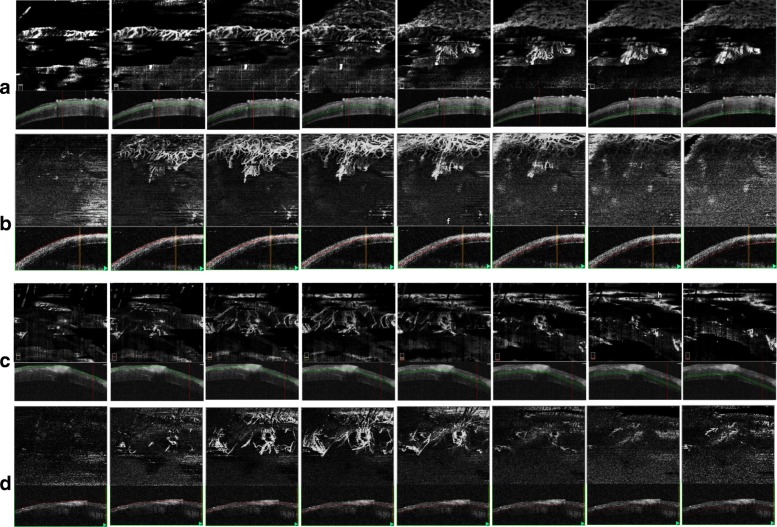
Comparison of En-face OCTA segmented images. The en-face and corresponding cross-sectional segmentation performed at every 50 μm depth in the SSADA OCTA system (Fig. 2**a**) versus the OMAG OCTA system (Fig. 2**b**) for the OCTA images in Fig. 1a imaged at the Week 2 follow-up time-point is illustrated. Similar segmentation profile for Fig. 1b at Week 1 follow-up time point is shown as Fig. 2**c** using in SSADA OCTA and Fig. 2**d** with OMAG OCTA. The segmentation lines in SSADA cross-sectional B-scans are marked in green, whereas in OMAG B-scans they are highlighted in red. The existence of vessels in the deeper layer segmented en-face images with the SSADA OCTA as projected from the superficial layers is observed in Fig. 2**a** and **c**

Representative OCTA images captured during Week 1 and Week 2 time-points at the same regions of interests are shown in Fig. 1. The same representative images segmented at every 50 μm of the cornea B-scan were extracted from each OCTA volume in the two systems [8, 19] and compared as shown in Fig. 2. The segmentation algorithm that was incorporated in the SSADA system was based on the macula B-scan layer segmentations, whereas the OMAG OCTA volume segmentation was based on the custom parallel layer segmentation developed for research purposes.

### Image processing

**Fig. 3:**
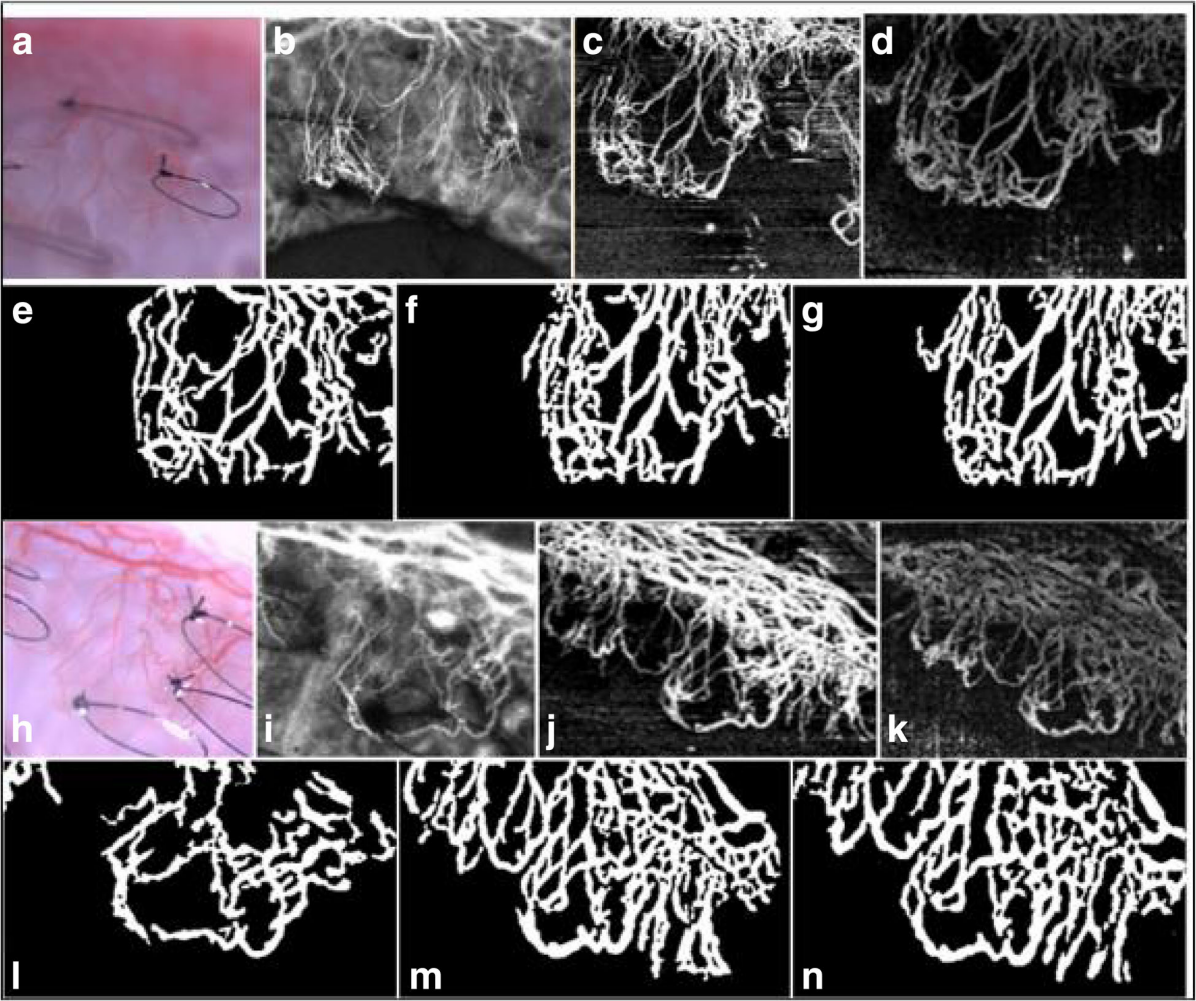
Illustrative examples of slit lamp photography (SLP), indocyanine green angiography (ICGA) and optical coherence tomography (OCTA) imaged for Vessel Analysis. (Top row) Examples of (**a**) SLP, (**b**) ICGA, (**c**) OMAG OCTA and (**d**) SSADA OCTA images imaged at the same ROI in the rabbit cornea at the week 2 time-point. (Second row) Binarized pictures of the corresponding (**e**) ICGA, (**f**) OMAG OCTA and (**g**) SSADA OCTA from the first row. (Third row) Examples of (**h**) Slit lamp, (**i**) ICGA, (**j**) OMAG OCTA and (**k**) SSADA OCTA images taken on rabbit 5 at the week 1 time-point. (Last row) Binarized images from third row sequence of (**l**) ICGA, (**m**) OMAG OCTA and (**n**) SSADA OCTA, respectively

All image processing was performed using MATLAB R2017b (The MathWorks, Inc., Natick, Massachusetts, United States) similar to a previously described technique [5]. OCTA images from the SSADA system were extracted in the Portable Network Graphics and Bitmap image file formats from the OMAG system. ICGA images were extracted in the Joint Photographic Experts Group format. Briefly, the extracted images from the three systems were first automatically registered for matching overlap in the captured region of area. Thereafter, filters were applied to remove speckle and motion artefacts. Following which, binarization using Otsu’s method of intensity threshold based on automatic binarization-level decisions was performed, wherein white and black pixels represented the vasculature foreground and the background, respectively. Figure 3 shows an illustrative example of binarized vessels performed in the processed images. Vessel density values were then computed from the binarized image as a ratio of the area of the white pixels (vessels) to the whole image pixel area.

### Statistical analysis

Statistical analysis was performed using MedCalc statistical software version 18.6 (MedCalc Software, Mariakerke, Belgium). For image quality comparison between the two OCTA type scan data, the Cohen’s kappa coefficient was calculated to measure intra-observer (comparison of image quality scores from the two different OCTA systems) and inter-observer (comparison of image quality scores by the two observers) agreement. Image quality scores were standardized to range from 0 to 4, where score 4 indicated very good quality and score 0 very poor quality. The kappa value was standardized to lie on a − 1 to 1 scale where 1 is perfect agreement and 0 represents what would be expected by chance. Negative values indicate potential disagreement between the observers [20]. Comparison between the vessel densities of SSADA OCTA, OMAG OCTA and ICGA processed images was computed using the paired t-test. Pearson correlation coefficient (*r* value) was used to determine the correlation between vessel density measurements of SSADA OCTA, OMAG OCTA and ICGA. Bland-Altman plots was evaluated to analyse the agreement between the three techniques; the difference of vessels’ density measurements between the imaging modalities was plotted against the average vessels’ density measurements of the methods. Further, vessel density values from the segmented en-face images using the two OCTA systems were also subjected to the Bland-Altman plot to show the different score measurements at the various depth segmentation ranges.

## Results

Overall, the vessel density values comparing the two OCTA techniques and ICGA generally showed agreement. Using the paired t-test, it was shown that measurement comparisons between SSADA OCTA and OMAG OCTA (*p* = 0.925), SSADA OCTA and ICGA (*p* = 0.332), and OMAG OCTA and ICGA (*p* = 0.500) showed no significant difference and had good correlation values (*r* > 0.9). In comparing the values acquired with the three imaging techniques, the ICGA vasculature (μ = 16.52 ± 8.94%) was observed to be more comparable to the OMAG OCTA (μ = 16.23 ± 9.51%) system than the SSADA OCTA system (μ = 17.09 ± 7.34%). Although we observed that the SSADA OCTA images (2.5, 2.0–4.0) rendered smoother images with lesser speckle and grey noise than the OMAG OCTA images (2.0, 2.0–3.0), the image quality scores were found to be comparable (*p* = 0.076) with good inter-observer agreement (κ = 0.704). Using Fig. 3 as a representative example, the higher vessel density observation in OCTA is demonstrated due to its ability to capture more vessels than slit lamp photography or ICGA. As SLP and ICGA have limited lateral resolution, this could potentially explain the reason for their reduced vascular acquisition. Table 1 lists the vessel density percentages computed from the 32 sets of matched images.

**Table 1:** Vessel density measurements computed from ICGA and OCTA in 16 rabbits at two consecutive follow-up time-points

Serial No.	ICGA Vessel Density (%) (μ = 16.52 ± 8.94%)	OMAG OCTA Vessel Density (%) (μ = 16.23 ± 9.51%)	SSADA OCTA Vessel Density (%) (μ = 17.09 ± 7.34%)
1	21.49	18.37	18.92
2	20.32	14.72	15.94
3	18.19	23.03	22.35
4	21.92	23.85	22.11
5	22.18	20.08	23.15
6	28.11	30.07	28.93
7	32.53	39.71	26.31
8	17.04	17.69	18.80
9	37.00	36.74	29.66
10	17.96	16.24	23.49
11	18.82	19.26	19.84
12	23.42	27.63	25.51
13	26.50	22.41	23.23
14	24.79	24.24	27.31
15	27.21	29.83	21.06
16	30.19	20.91	29.01
17	6.72	6.46	6.87
18	5.44	5.61	6.20
19	7.62	5.84	8.84
20	7.93	8.43	10.45
21	7.73	6.66	9.41
22	11.41	8.44	12.96
23	8.69	7.14	11.97
24	11.81	9.55	10.01
25	8.39	10.26	11.95
26	14.87	14.40	16.65
27	7.54	8.34	10.36
28	10.84	8.82	12.76
29	8.68	11.55	12.09
30	6.80	8.25	10.50
31	10.20	9.03	13.96
32	6.32	5.71	6.41

**Fig. 4:**
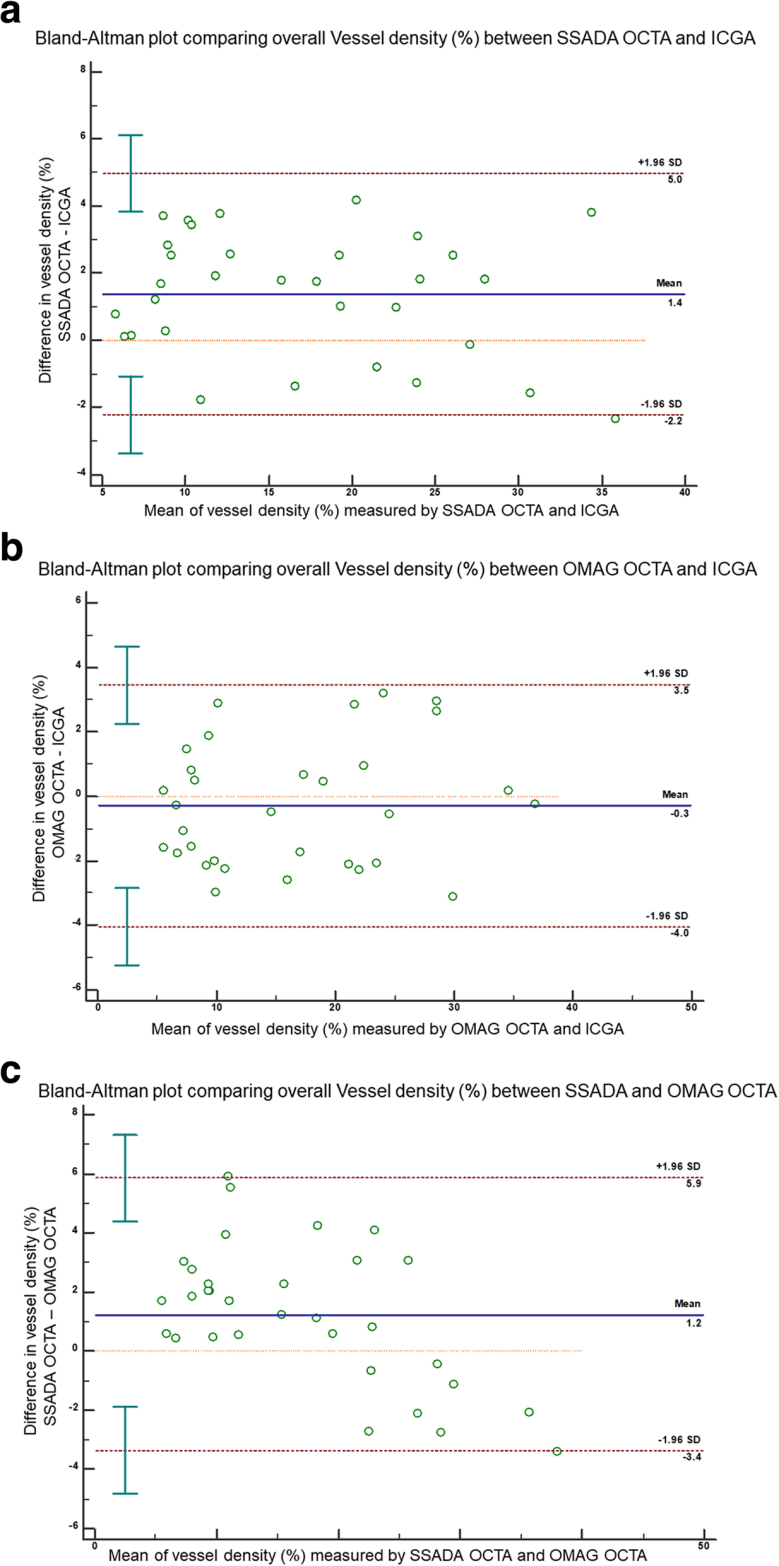
Bland-Altman plots comparing vessels density measurements from OCTA with ICGA. The Bland-Altman plot between the differences (y-axis) of vessels density measurements from (**a**) SSADA OCTA and ICGA, (**b**) OMAG OCTA and ICGA and (**c**) SSADA OCTA and OMAG OCTA as the deviation from the mean vessels density values comparing the corresponding two methods (x-axis) — showing good agreement of vessels density between all imaging methods. Solid line = mean of the difference. Short dashed line = reference zero. Long dashed line = upper and lower 95% limits of agreement (mean + 1.96 SD, mean − 1.96 SD). SD = standard deviation of the mean difference

Figure 4 demonstrates the Bland-Altman plots agreements comparing the angiography techniques computed from the 32 sets of matched images. There was good agreement between all three imaging modalities in terms of vessel density measurements. ICGA vs. SSADA OCTA (*r* > 0.7) LOA lower limit − 15.44 μm (95% CI: − 20.072 to − 10.825 μm); upper limit 13.657 μm (95% CI: 9.033 to 18.280 μm); ICGA vs. OMAG OCTA (*r* > 0.9) LOA lower limit − 9.713 μm (95% CI: − 12.547 to − 6.880 μm); upper limit 8.125 μm (95% CI: 5.292 to 10.959 μm); SSADA OCTA vs. OMAG OCTA (r > 0.7) LOA lower limit − 12.585 μm (95% CI: − 16.550 to − 8.619 μm); upper limit 12.381 μm (95% CI: 8.415 to 16.347 μm).

**Fig. 5:**
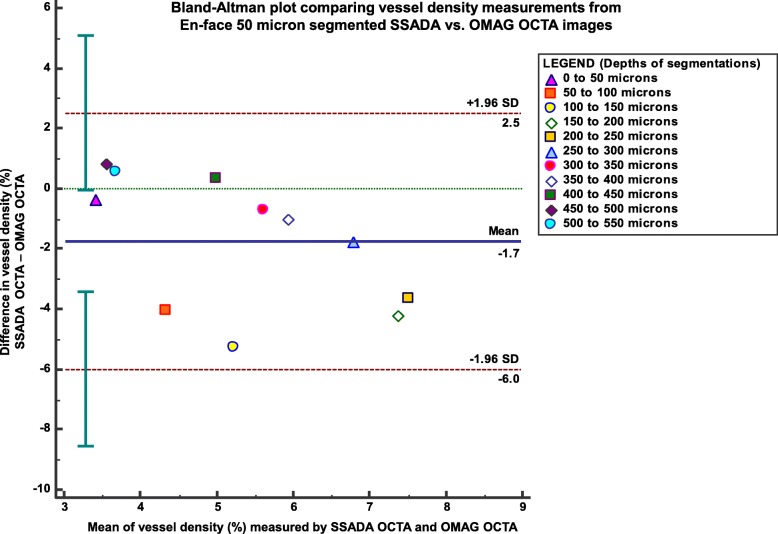
Vessel density measurements compared at every 50-μm segmentation depth between OMAG OCTA and SSADA OCTA. Bland-Altman plot comparing vessel density measurements between OMAG OCTA and SSADA OCTA. Each marker represents the average measurement at each segmentation depth measured from 10 sets of matched segmented images. Solid line = mean of the difference. Short dashed line = reference zero. Long dashed line = upper and lower 95% limits of agreement (mean + 1.96 SD, mean − 1.96 SD). SD = standard deviation of the mean difference

We also obtained a good correlation value (*r* = 0.993) when comparing vessel density measurements of the en-face segmentations at every 50 μm between the OCTA systems. In superficial depth segmentations, the OMAG OCTA provided higher vessel density values than the SSADA OCTA system (mean vessel density 6.172 ± 3.6% vs. 4.377 ± 2.2%, respectively, *p* < 0.001). However, in segmentation layers greater than 400 μm deep, the SSADA OCTA system mean vessel density measurements were higher (4.438 ± 2.127%) compared with the other system (4.041 ± 1.803%). The difference in the trend of vascular densities captured from the two devices is also shown in Fig. 2 as a representative example. In the last few segmentation depths of the SSADA OCTA system it is seen that it additionally captures the projection from the superficial segmentations. The vessel density extracted from each of the depth range segmentations from the two OCTA systems were plotted for their difference scores as a Bland-Altman graph as shown in Fig. 5. Good agreement between OMAG OCTA and SSADA OCTA was observed with a mean difference of 1.872 ± 1.942% (95% CI: 1.956 to 7.473%), *P* = 0.218.

## Discussion

Using the SSADA and OMAG algorithm-based OCTAs, we have demonstrated that the visualization of both dense and fine vasculature across the entire cornea are comparable to ICGA circulations without significant differences. It was observed that in the SSADA implemented images of SSADA OCTA, less axial bulk noise and smoother signal was present as compared to the OMAG algorithm-derived OCTA images. This observation can be attributed to the SSADA OCTA’s volume averaged acquisition from two repeated consecutive B-scans (each taking 3–4 s) with inbuilt motion correction software, as compared to the OMAG OCTA system that takes 5–6 s for one full scan, resulting in more motion artefacts in subjects with poor fixation [21]. Further, the improved signal-to-noise ratio could also be a result of the system being independent of phase information and is thereby insensitive to phase noise, giving rise to better signal strength. However, this advantage is also at the expense of the degradation of its axial resolution equalled to its transverse dimension, which can introduce undesirable projection artefacts [22].

This drawback of projection flow from superficial to deeper layers contributes to inaccuracy in en-face projections of the SSADA system for the reconstruction of deeper layer vasculature segments [18]. This limitation of the SSADA system is a possible reason for the observation of significantly higher vessel density values in the SSADA system than the OMAG system in the deeper layers of the cornea. On the other hand, the OMAG OCTA system overcomes these limitations of the SSADA system as it is not associated with projection artefact issues. The system processes images using both phase and amplitude information, using the algorithm of complex OCT signal difference (CODAA). This allows for the additional inclusion of flow-induced changes from the phase of OCT signal, thus providing ultra-high sensitivity for the detection of micro-circulations [17, 23]. The phase variance method, which is known to be the best method amongst all others to offer good contrast-to-noise ratio, also allows for effective suppression of the static tissue noise [21]. These strengths of the CODAA system support our findings that vessel density values of the ICGA images are more comparable to the OMAG OCTA system than the SSADA system. In overall comparison to ICGA, it was generally observed that the OMAG OCTA provided better quantitative agreement and the SSADA OCTA showed slightly better performance in quality.

In the second part of the analysis in the study, we reported for the first time the comparisons of en-face segmentations at every 50 μm between two anterior segment OCTA systems. The vessel density measurements at all depth segmentations correlated well in both systems, with no significant difference. It was observed that the OMAG OCTA system was found to have higher vessel density measurements than the SSADA OCTA system, in segmentation depth ranging from 0 to 350 μm. This may be because of the higher contrast and working wavelength exhibited by the OCTA system. Conversely, in deeper segmentation layers (> 400 μm), the SSADA system over-estimated the measurement, which could have been associated with the inaccuracy of vessel density projections from the more superficial layers. Although the three-dimensional en-face scan tomography provided reasonable and reliable segmentation profiles for the cornea analysis, it should be noted that the extracted image outcomes may not be precise as they were not based on segmentation algorithms developed for anterior-segment B-scans and is less robust [13]. Non-parallel segmentations and layer identification artefacts may contribute to errors in the en-face segmentations, especially in poor quality OCTA scans, where the segmentation lines were not oriented parallel to the corneal surface [15].

As a result, despite our study showing direct comparisons of the two OCTA systems for the same regions in the same subjects, factors associated with differences in segmentation and acquisition protocols in the two OCTA systems may not account for one-to-one comparison of their performances and analyses. Furthermore, device-dependent parameters such as the difference in speed, operating wavelengths, contrast-to-noise ratio, signal-to-noise ratio and sensitivity were not taken considered while comparing the vessel density outcomes processed from the two OCTA systems [13].

Therefore, we observed that there is generally a better agreement of the OMAG OCTA system to ICGA. However, it cannot be conclusively decided that the OMAG OCTA system performs better than the SSADA system. Both the OCTA systems are found to be comparable to the ICGA imaging system to image the vasculature in the anterior-segment eye and are associated with their respective advantages and limitations based on their implemented algorithm. For example, the amplitude decorrelated images obtained from the SSADA system, provided a better signal-to-noise ratio, but was susceptible to bulk tissue motion noise and projection artefacts [18]. On the other hand, while the phase-variance method employed in the OMAG system provided higher sensitivity to vascular details and independence of projection artefacts, it was still subject to greater background noise and motion artefacts. It is important to note that these findings are relevant in the case of optimal operation of the system comparable to the animal model setting where there is control of eye movements and limited motion artefacts present. In the clinical setting, the quality of the images and volume of artefacts may vary considerably.

## Conclusion

In this experimental study, we compared and validated two OCTA systems with ICGA to delineate corneal vessels in an animal model. The overall vessel density measurements for both systems were comparable to the ICGA technique, where there was less difference between ICGA and OMAG OCTA than ICGA and SSADA OCTA system in the same region of corneal vascularisation. The en-face segmentation analysis of the two systems showed that the SSADA OCTA relative to the OMAG OCTA under-estimated vessel density in the superficially segmented angiography layers whereas, the OMAG OCTA under-estimated the same in deeper vasculature layers. Future studies are required to validate the differences between OCTA systems with histology, compare for repeatability assessments and use segmentation algorithms implemented for the cornea. With OCTA technology advancing at a rapid rate than the community’s experience with the technique, the need for the standardisation of anterior segment protocols and accurate segmentation software across competing OCTA technologies for its image acquisition and interpretation is demanding. Evaluation of OCTA into a multimodal platform alongside other established imaging techniques will provide us with a better understanding to correctly assess the vasculature of the cornea and ocular surface diseases. This will enable the advancement of OCTA into clinical practice as a more precise and efficient diagnostic modality for the cornea.

## Abbreviations

CI: Confidence interval
ICG: Indocyanine green
ICGA: Indocyanine green angiography
LOA: Limits of agreement
OCT: Optical coherence tomography
OCTA: Optical coherence tomography angiography
OMAG: Optical micro-angiography
SD: Spectral domain
SLP: Slit lamp photography
SSADA: Split-spectrum amplitude decorrelation angiography
