# Correction to: Vessel density and En-face segmentation of optical coherence tomography angiography to analyse corneal vascularisation in an animal model

**DOI:** 10.1186/s40662-019-0132-7

**Published:** 2019-02-14

**Authors:** Kavya Devarajan, Wen Di Lee, Hon Shing Ong, Nyein C. Lwin, Jacqueline Chua, Leopold Schmetterer, Jodhbir S. Mehta, Marcus Ang

**Affiliations:** 1 Singapore Eye Research Institute, Singapore, Singapore; 2 Singapore National Eye Center, Singapore, Singapore; 3 Eye-ACP, Duke-NUS Graduate Medical School, Singapore, Singapore; 4 Department of Clinical Pharmacology, Medical University of Vienna, Vienna, Austria; 5 Center for Medical Physics and Biomedical Engineering, Medical University of Vienna, Vienna, Austria; 6 Nanyang Technological University, Singapore, Singapore

**Correction to: Eye Vis (2019) 6:2**

**https://doi.org/10.1186/s40662-018-0128-8**

In the original publication of this article [1] the algorithm of the OCTA (Nidek RS-3000) was described incorrectly as OMAG (Optical micro angiography). However, the system uses CODAA(Complex OCT signal difference angiography). Thus, ‘OMAG’ in the article should be replaced with ‘CODAA’, and ‘Optical micro angiography’ should be replaced with ‘Complex OCT signal difference angiography’!
